# Supplementary figures and images for: Screening and verification of reference genes for analysis of gene expression in winter rapeseed (Brassica rapa L.) under abiotic stress
Source: PLoS One. 2020 Sep 17;15(9):e0236577. doi: 10.1371/journal.pone.0236577 (PMC7498103; doi:10.1371/journal.pone.0236577)

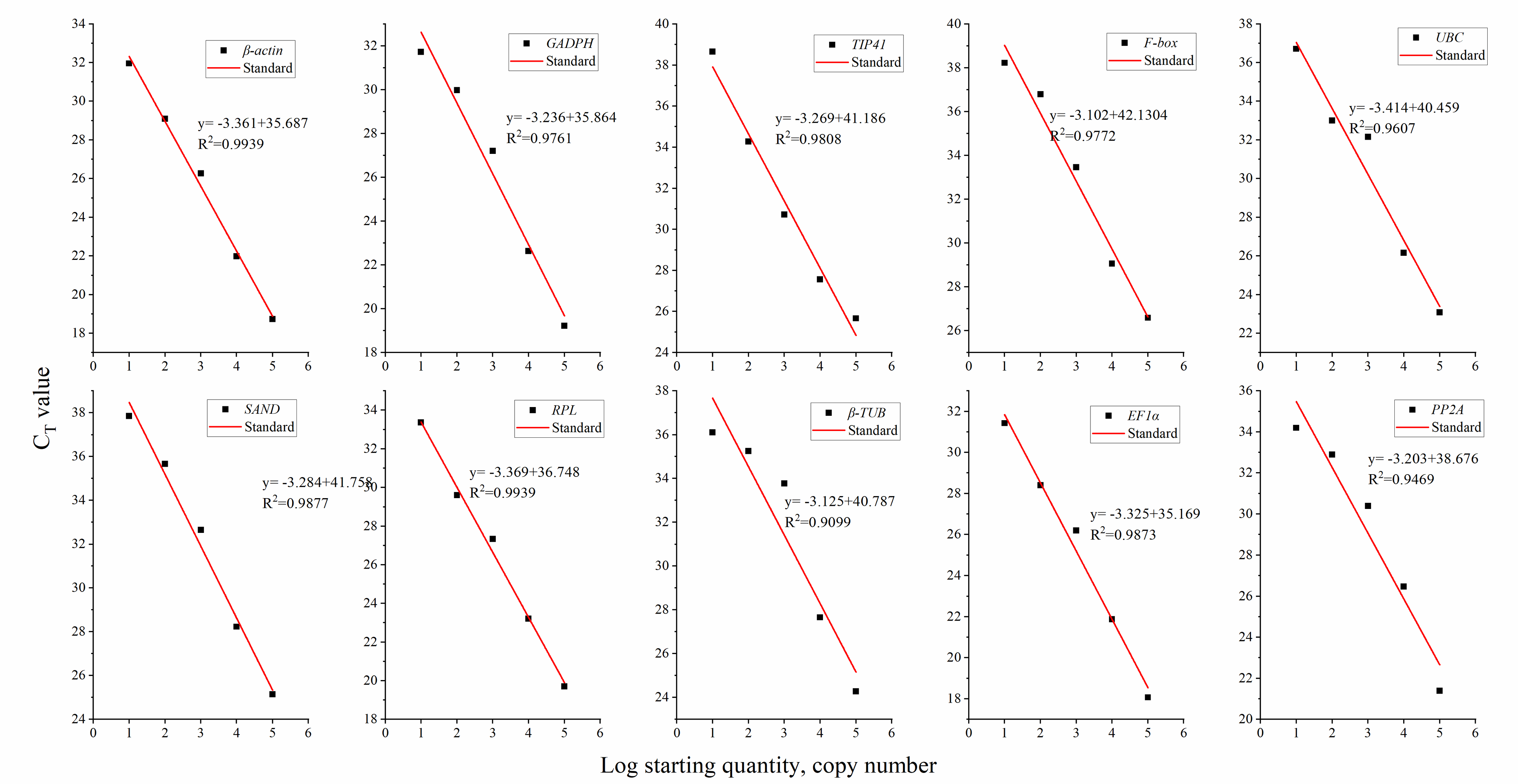

Supplement: S1 Fig — (TIF) [file pone.0236577.s001.tif]

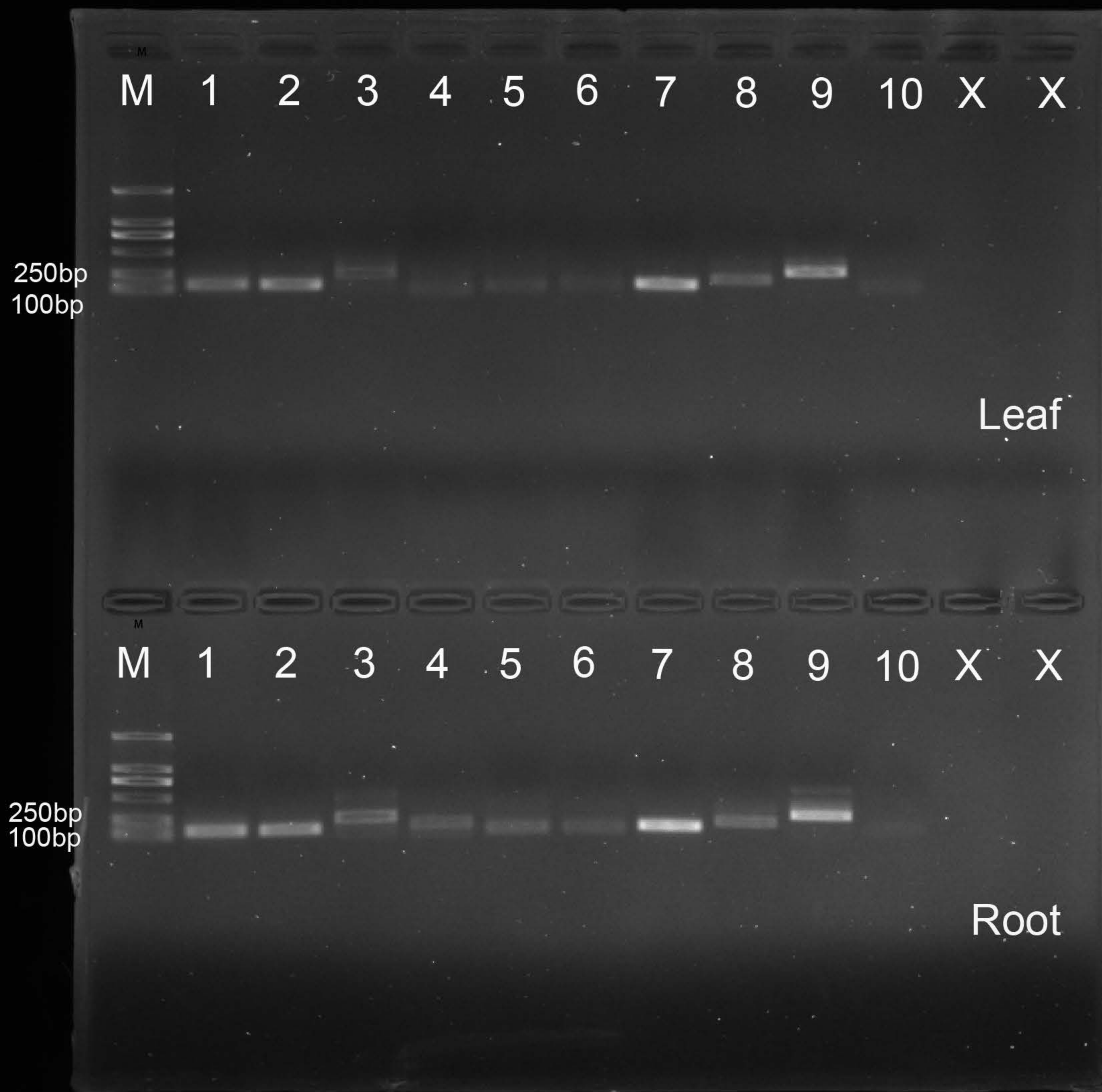

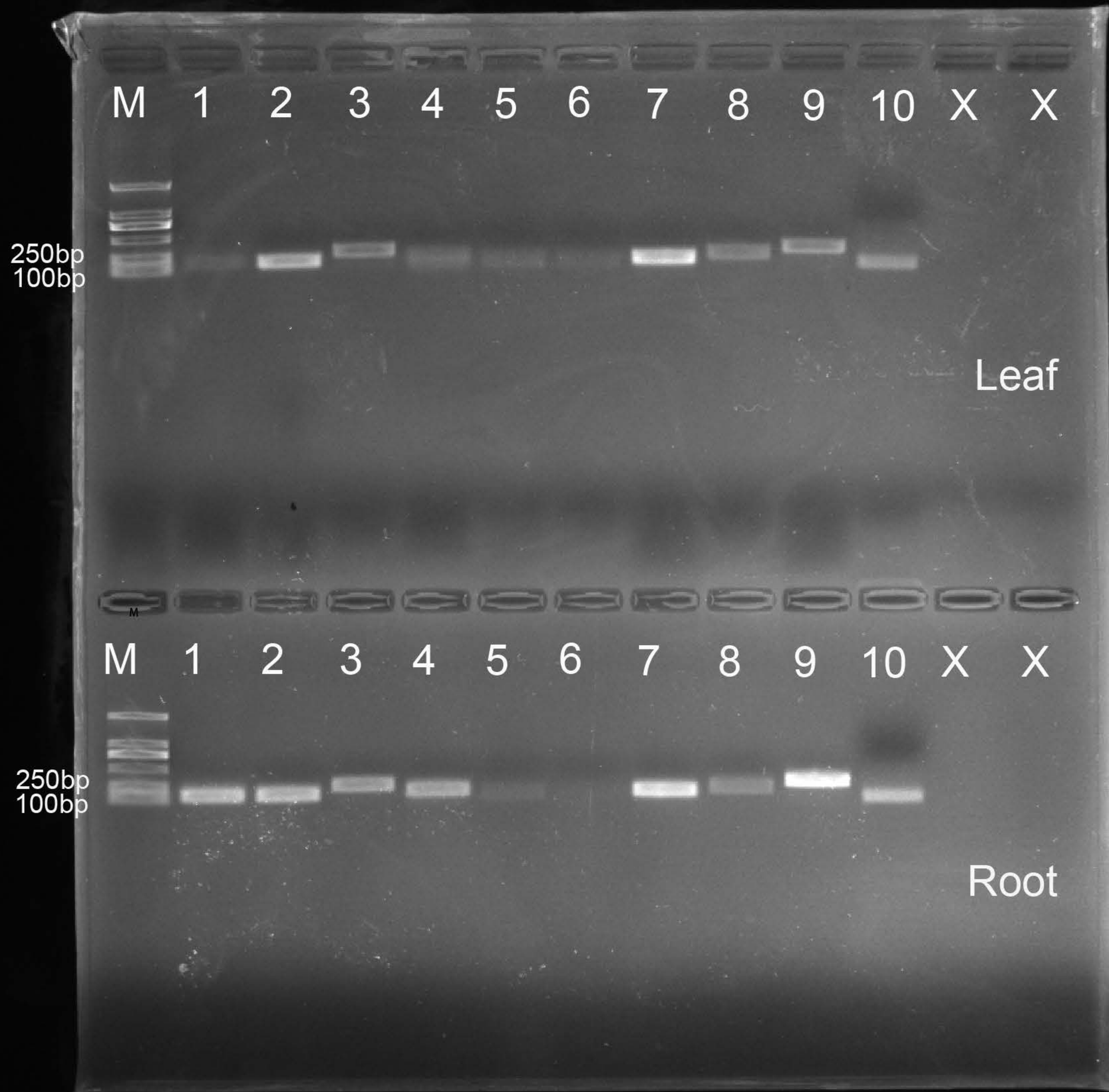

250bp  
100bp

M 1 2 3 4 5 6 7 8 9 10 X X

Leaf

250bp  
100bp

M 1 2 3 4 5 6 7 8 9 10 X X

Root

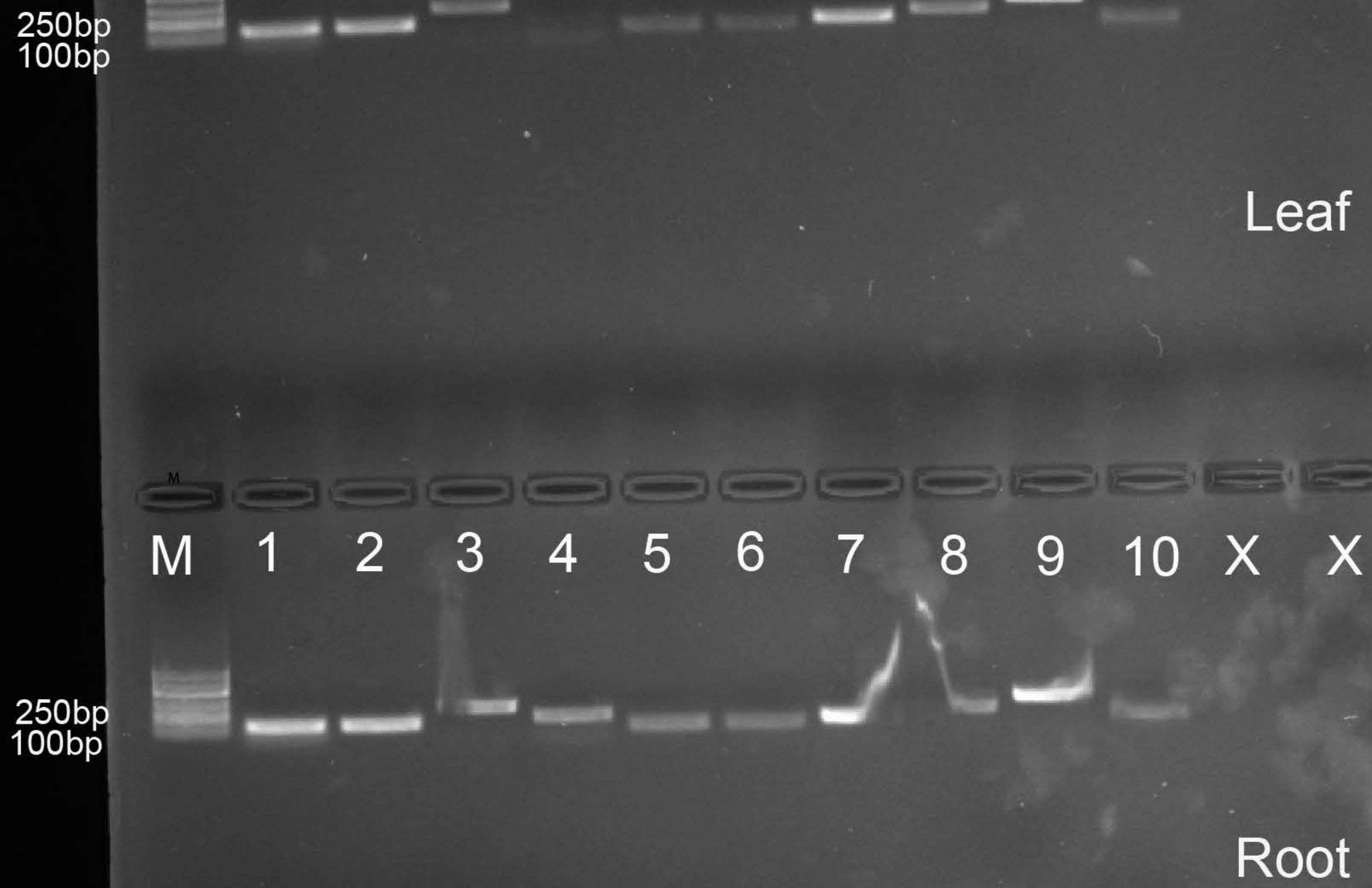

Supplement: S1 Raw images — (PDF) [file pone.0236577.s008.pdf]
