# Supplementary material for: Screening and verification of reference genes for analysis of gene expression in winter rapeseed (Brassica rapa L.) under abiotic stress
Source: PLoS One. 2020 Sep 17;15(9):e0236577. doi: 10.1371/journal.pone.0236577 (PMC7498103; doi:10.1371/journal.pone.0236577)
Supplement: Attachment — Submitted filename: Response to Reviewers.docx [file pone.0236577.s010.docx]

Response to Reviewer #1:

1- Authors did not mentioned qPCR conditions in material and methods.

**Response 1:** The reaction conditions of qPCR have been supplemented in material and methods.

2- A sequence of PCR product is also needed in order to confirm that the specific gene has been amplified.

**Response 2:** We rechecked 10 gene sequences with primers, A single band was obtained by PCR amplification with primers, and the specific sequence was obtained by sequencing. Here is the result.

*β-actin*: Sorry, Professor, we sequenced the actin primers to get a single strip and sequencing result. The comparison result is shown in the figure below. After NCBI comparison, it shows that it is beta actin 2 gene (XM_018658258.2), and the primer information has been updated.

*GADPH*: No paralogs with high sequence identities, and NCBI has provided a new version of this gene, which I have updated in materials and methods.

*TIP41*: A single band was obtained by PCR amplification with primers, and the specific sequence was obtained by sequencing. And NCBI has provided a new version of this gene, which I have updated in materials and methods.

*F-box*: A single band was obtained by PCR amplification with primers, and the specific sequence was obtained by sequencing. And NCBI has provided a new version of this gene, which I have updated in materials and methods.

*UBC*: A single band was obtained by PCR amplification with primers, and the specific sequence was obtained by sequencing. And NCBI has provided a new version of this gene, which I have updated in materials and methods.

*SAND*: A single band was obtained by PCR amplification with primers, and the specific sequence was obtained by sequencing. And NCBI has provided a new version of this gene, which I have updated in materials and methods.

*RPL*: A single band was obtained by PCR amplification with primers, and the specific sequence was obtained by sequencing. And NCBI has provided a new version of this gene, which I have updated in materials and methods.

*β-TUB*: A single band was obtained by PCR amplification with primers, and the specific sequence was obtained by sequencing. And NCBI has provided a new version of this gene, which I have updated in materials and methods.

*EF1α*: A single band was obtained by PCR amplification with primers, and the specific sequence was obtained by sequencing. And NCBI has provided a new version of this gene, which I have updated in materials and methods.

*PP2A*: A single band was obtained by PCR amplification with primers, and the specific sequence was obtained by sequencing. And NCBI has provided a new version of this gene, which I have updated in materials and methods.

3- Please avoid some unnecessary some space in sentences and typing errors

For example line 52, 54, 56, 76 and 77 have spacing problem.

Line 26 there you typed EF-1a instead of EF1α.

Line 114 you added 2.4 as a bullet numbering.

**Response 3:** These errors have been corrected in the manuscript and the full text examined carefully.

Response to Reviewer #2:

I am sorry to say that author did not consider my first comments. I have checked most genes whether they have multigenes showing high identities. Except TIP4l, most genes have more than two paralogs with high sequence identities. Therefore, authors should check whether primer sequences shown in Table 1 is specific for one of them or common. XM_009127096.2 is beta-actin 7 gene, but it does not have primer sequences in Table 1. Instead, beta-actin 2 gene contains forward primer, but no reverse primer sequence at all. Please check, specify primer and paralog gene.

**Response Reviewer #2:** Sorry, Professor, I didn't understand your question and didn't answer it completely in the first reply. This time, We rechecked 10 gene sequences with primers, and sequenced them after PCR amplification to ensure that the primers were specifically amplified. Here is the result.

*β-actin*: Sorry, Professor, we sequenced the actin primers to get a single strip and sequencing result. The comparison result is shown in the figure below. After NCBI comparison, it shows that it is beta actin 2 gene (XM_018658258.2), and the primer information has been updated.

*GADPH*: No paralogs with high sequence identities, and NCBI has provided a new version of this gene, which I have updated in materials and methods.

*TIP41*: A single band was obtained by PCR amplification with primers, and the specific sequence was obtained by sequencing. And NCBI has provided a new version of this gene, which I have updated in materials and methods.

*F-box*: A single band was obtained by PCR amplification with primers, and the specific sequence was obtained by sequencing. And NCBI has provided a new version of this gene, which I have updated in materials and methods.

*UBC*: A single band was obtained by PCR amplification with primers, and the specific sequence was obtained by sequencing. And NCBI has provided a new version of this gene, which I have updated in materials and methods.

*SAND*: A single band was obtained by PCR amplification with primers, and the specific sequence was obtained by sequencing. And NCBI has provided a new version of this gene, which I have updated in materials and methods.

*RPL*: A single band was obtained by PCR amplification with primers, and the specific sequence was obtained by sequencing. And NCBI has provided a new version of this gene, which I have updated in materials and methods.

*β-TUB*: A single band was obtained by PCR amplification with primers, and the specific sequence was obtained by sequencing. And NCBI has provided a new version of this gene, which I have updated in materials and methods.

*EF1α*: A single band was obtained by PCR amplification with primers, and the specific sequence was obtained by sequencing. And NCBI has provided a new version of this gene, which I have updated in materials and methods.

*PP2A*: A single band was obtained by PCR amplification with primers, and the specific sequence was obtained by sequencing. And NCBI has provided a new version of this gene, which I have updated in materials and methods.
